# A review of machine learning models applied to genomic prediction in animal breeding

**DOI:** 10.3389/fgene.2023.1150596

**Published:** 2023-09-06

**Authors:** Narjice Chafai, Ichrak Hayah, Isidore Houaga, Bouabid Badaoui

**Affiliations:** ^1^ Laboratory of Biodiversity, Ecology, and Genome, Department of Biology, Faculty of Sciences, Mohammed V University in Rabat, Rabat, Morocco; ^2^ Centre for Tropical Livestock Genetics and Health, The Roslin Institute, Royal (Dick) School of Veterinary Medicine, The University of Edinburgh, Edinburgh, United Kingdom; ^3^ The Roslin Institute, Royal (Dick) School of Veterinary Studies, University of Edinburgh, Edinburgh, United Kingdom; ^4^ African Sustainable Agriculture Research Institute (ASARI), Mohammed VI Polytechnic University (UM6P), Laayoune, Morocco

**Keywords:** artificial intelligence, algorithms, classification, regression, genomic selection, animal breeding, SNPs

## Abstract

The advent of modern genotyping technologies has revolutionized genomic selection in animal breeding. Large marker datasets have shown several drawbacks for traditional genomic prediction methods in terms of flexibility, accuracy, and computational power. Recently, the application of machine learning models in animal breeding has gained a lot of interest due to their tremendous flexibility and their ability to capture patterns in large noisy datasets. Here, we present a general overview of a handful of machine learning algorithms and their application in genomic prediction to provide a meta-picture of their performance in genomic estimated breeding values estimation, genotype imputation, and feature selection. Finally, we discuss a potential adoption of machine learning models in genomic prediction in developing countries. The results of the reviewed studies showed that machine learning models have indeed performed well in fitting large noisy data sets and modeling minor nonadditive effects in some of the studies. However, sometimes conventional methods outperformed machine learning models, which confirms that there’s no universal method for genomic prediction. In summary, machine learning models have great potential for extracting patterns from single nucleotide polymorphism datasets. Nonetheless, the level of their adoption in animal breeding is still low due to data limitations, complex genetic interactions, a lack of standardization and reproducibility, and the lack of interpretability of machine learning models when trained with biological data. Consequently, there is no remarkable outperformance of machine learning methods compared to traditional methods in genomic prediction. Therefore, more research should be conducted to discover new insights that could enhance livestock breeding programs.

## 1 Introduction

Farmers and animal breeders have long used artificial selection to produce offspring with specific desired traits. Assessing the performance of animals was based solely on phenotypes for centuries; it was not until the 20th century that pedigree records and performance data became the keys to genetic selection programs (Boichard et al., 2016). Several statistical methods were developed to predict the breeding values of individuals, such as selection index and Mixed Model Equations (MME), which allowed, due to advances in computational power, the Best Linear Unbiased Prediction (BLUP) (Henderson, 1984) to become the most sophisticated approach for breeding value estimation and thus enable accurate selection decisions (Meuwissen et al., 2016). Nevertheless, traditional genetic evaluation techniques are generally more reliable in estimating breeding values for phenotypic traits that can be easily measured and have moderate to high heritability (Boichard et al., 2016). Conversely, traits with low heritability necessitate a substantial quantity of pedigree and phenotype data, which increases the generation interval and subsequently diminishes the overall genetic improvement accomplished through the breeding program. The emergence of molecular genetics has prompted researchers to delve into a comprehensive investigation of how traits are determined at the DNA level. Numerous studies have been carried out with the aim of pinpointing particular segments within the genome that play a crucial role in accounting for variations in genetic characteristics known as Quantitative Trait Loci. Later in the 1980s to the 2000s, several methods were proposed for marker-assisted selection (MAS) research that incorporate information about QTL in the MME as fixed effects, and thus breeding value estimation is performed by summing the estimated effects for every QTL (Weigel et al., 2017). Nevertheless, the effectiveness of incorporating Quantitative Trait Loci into estimating breeding values was constrained by the sparse distribution of markers that were in linkage disequilibrium with QTL across the entire population. Furthermore, it was discovered that quantitative traits are influenced by a multitude of QTL with relatively minor individual contributions. Meuwissen et al. (2001) proposed a multiple QTL methodology named genomic selection, that estimates breeding values using a dense marker map. Genomic selection assumes that estimating the effects of a large number of single nucleotide polymorphism (SNP) across the genome will enable breeding value estimation without prior knowledge of the location of specific genes on the genome (Eggen, 2012).

In 2007, progress in molecular technology allowed the first assembly of the bovine genome. The Illumina Company and an international consortium introduced a chip to genotype simultaneously over 54,000 SNPs, which revolutionized dairy cattle breeding (Boichard et al., 2016), and consequently, various methods were developed for whole-genome selection in plants and other domestic animal species. Recently, the availability of high-throughput genotyping and the decrease in genotyping costs have made genomic selection a standard method in animal breeding schemes in many countries (Meuwissen et al., 2016). The underlying concept is based on predicting markers effects using phenotypic information and the genomic relationship between individuals of a reference population previously genotyped and phenotyped to forecast the breeding values of a certain trait for a population of genotyped selection candidates (Goddard et al., 2010). Various statistical methods, such as Genomic Best Linear Unbiased Prediction (GBLUP) or Bayesian methods with different prior assumptions, have been developed to predict markers' effects and thus the genomic breeding values of individuals. Nevertheless, these conventional methods were unable to consider non-additive effects such as epistasis and interactions between genotypes (Bayer et al., 2021) which can have a large effect on phenotypes in animal species. Furthermore, genotyping provides ever-increasing marker datasets, which exacerbates the “curse of dimensionality” also known as the “large P, small N” paradigm (Nayeri et al., 2019). Consequently, traditional linear models became inadequate for capturing patterns and explaining the complex relationships hidden in this mass of large noisy data.

Recently, the development of machine learning (ML) algorithms and the concomitant boost in computational processing power have generated buzz in the scientific community. ML models are known for their tremendous flexibility and their ability to extract hidden patterns in large noisy datasets, such as image-based data (Xiao et al., 2015), massive datasets of heterogeneous records (Li et al., 2018b), or digital data, which is increasing remarkably due to advancements in computer vision, natural language processing (NLP), internet of things (IoT), or computer hardware (David et al., 2019). Genomics, due to the advent of sequencing technologies, became a field where researchers deal with massive, heterogeneous, redundant, and complex omics datasets. Thus, the application of machine learning models in genomics has been investigated in several studies. In this paper, we review the application of ML algorithms to genomic prediction (GP) in livestock breeding. This work is organized as follows: First, we discuss machine learning fundamentals and provide a brief description of common algorithms used in genomic prediction. Second, we outline the different evaluation methods used to assess the performance of ML models. Afterwards, we review some of the published studies concerning the application of ML models in genomic prediction to provide a meta-picture of their potential in terms of prediction accuracy and computational time. Finally, we discuss the potential of applying ML to animal breeding in low- and middle-income countries.

## 2 Machine learning fundamentals

Machine learning can be defined as a branch of artificial intelligence that empowers computer systems to learn without being voraciously programmed (Sharma and Kumar, 2017). In other words, a learning computer system can be described as a computer whose performance P on task T improves as its experience E increases (Kang and Jameson, 2018). Based on the learning process, machine learning algorithms can be classified into supervised learning, unsupervised learning and reinforcement learning.

### 2.1 Supervised learning

In supervised learning, the learning process consists of conceiving a meaning from labeled data. Mainly, supervised learning algorithms tend to estimate or predict a response variable y , based on a set of explicative variables x , through a function called *predictor* $f\left(x,\beta \right)$ where β is a vector of model parameters. The performance criterion we use to define the best *predictor* is called a *loss function* L *,* we thus define the best predictor as the predictor who minimizes the loss function L (Crisci et al., 2012; Pereira and Borysov, 2019). Depending on the nature of the response variable y (continuous or discrete), supervised learning algorithms are applied to either regression or classification problems. If the main task of an algorithm is to predict a numeric value of a continuous target variable, the ML algorithm performs a regression problem. Alternatively, a classification problem consists of training the algorithm using a set of labeled features (discrete variable), to learn how to successfully classify new features accordingly (Kang and Jameson, 2018). Sometimes the training data involves labeled and unlabeled data. This type of learning is called semi-unsupervised learning and it is considered a class of supervised learning tasks. Anomaly detection is a typical application of semi-supervised learning algorithms (Kang and Jameson, 2018).

### 2.2 Unsupervised learning

Unsupervised learning consists of finding patterns or clusters in the training data where the target variable is not present. Algorithms learn on their way to discovering interesting structures in the training data (Mahesh, 2020). Since the features fed to the algorithms are unlabeled, there is no way of assessing the accuracy of these algorithms, unlike supervised learning and reinforcement learning. These models are mainly used for clustering and feature reduction (Sharma and Kumar, 2017).

### 2.3 Reinforcement learning

In reinforcement learning, software agents perceive and interpret their environment, perform actions and get rewards or penalties in return. Explicitly, a reinforcement learning algorithm enables an agent connected to its environment, to choose an action $a_{1}$ and generate an output y , given an input i and an environment $s_{1}$ . The action changes the environment, and a value is attributed to the transition of the environment’s state through a scalar reinforcement signal r . Consequently, the agent chooses actions that increase the sum of values of the reinforcement signal (Kaelbling et al., 1996). Similar to biological systems, animals living in specific environments face fundamental challenges such as locating sustenance, avoiding harm, and reproducing. These environmental conditions are subject to dynamic changes and sudden variations. Consequently, animals must continuously acquire knowledge from their surroundings and adapt their behaviors accordingly (Neftci and Averbeck, 2019). Similarly, when a robot is assigned the task of navigating a maze in reinforcement learning scenarios, it functions as an agent within this process. In its interactions with the maze environment, the robot seeks to identify optimal paths by taking successive actions (i.e., moving) while simultaneously receiving feedback through rewards for proximity to the exit or penalties for deviating further away or finding no escape route. By integrating these multiple-step feedback signals into its decision-making processes over time, the robot gradually enhances its navigation capabilities.

In the field of genomic prediction, supervised learning stands out as the most widely employed technique. This approach leverages labeled data to develop and assess models, thereby allowing for more direct predictions based on established patterns. In contrast, less prominence is given to unsupervised learning and reinforcement learning in relation to genomic prediction.

## 3 Common ML models used for genomic prediction

In the sections below, we present a short description of some widely used machine learning algorithms for genomic prediction.

### 3.1 Linear regression

Linear regression is a model usually used to forecast the value of a continuous variable y also called label or target variable using ML terminology, through a vector of explanatory variables also called independent variables or features X , and a linear function. If the model involves a single independent variable x , simple linear regression defines the relationship between the variables using the model:

$$y=\beta_{0}+\beta_{1}x+\varepsilon$$ (1)

where $\beta_{0}$ is the intercept term and $\beta_{1}$ is a regression coefficient that represents the variation in the outcome for a 1-unit increase in the value of the independent variable x , and ε represents the error term also called noise. The dependent variable y can be explained with more than one explanatory variable. In that case, we are talking about Multivariate Linear Regression (MLR). The basic model for MLR is Maulud and Abdulazeez (2020):

$$y=\beta_{0}+\beta_{1}x_{1}+...+\beta_{m}x_{m}+\varepsilon$$ (2)

Linear regression is considered a supervised learning algorithm because we feed the model with a data set containing features $x_{i}$ and the corresponding values of the target variable $y_{i}$ , and we expect an accurate prediction of $y_{j}$ for another set of features $x_{j}$ . In order to reach sufficient accuracy, the model minimizes the value of a chosen loss function (Nasteski, 2017). The most commonly used loss function for linear regression is Least Squared Error (LSE) (Maulud and Abdulazeez, 2020).

### 3.2 Logistic regression

Logistic regression is a classification model regularly applied for the analysis of dichotomous or binary outcomes (LaValley, 2008). In other words, logistic regression is used to study the effects of predictor variables on binary or categorical outcomes, such as the presence or absence of an event (Nick and Campbell, 2007). Training data is fed to a model that uses a logistic function in order to predict the probability of the event. Unlike linear regression, logistic regression does not require a linear relationship between dependent and independent variables, the model uses a log transformation to the odds ratio defined as the ratio of the probability of the event happening divided by the probability of the event not happening (LaValley, 2008). The logistic regression hypothesis is defined as (Nasteski, 2017):

$$h_{\theta }\left(x\right)=g\left(\theta^{T}x\right)$$ (3)

Where the function g is a sigmoid function defined as the following:

$$g\left(z\right)=\frac{1}{1+{\mathrm{e}}^{-z}}$$ (4)

Logistic regression uses a Maximum Likelihood Estimation (MLE) loss function, which is a conditional probability. The algorithm assigns each observation to class 0 or class 1 based on whether the probability is greater or smaller than a given threshold, 0.5 for example, (Belyadi and Haghighat, 2021).

### 3.3 Decision trees

**FIGURE 1:**
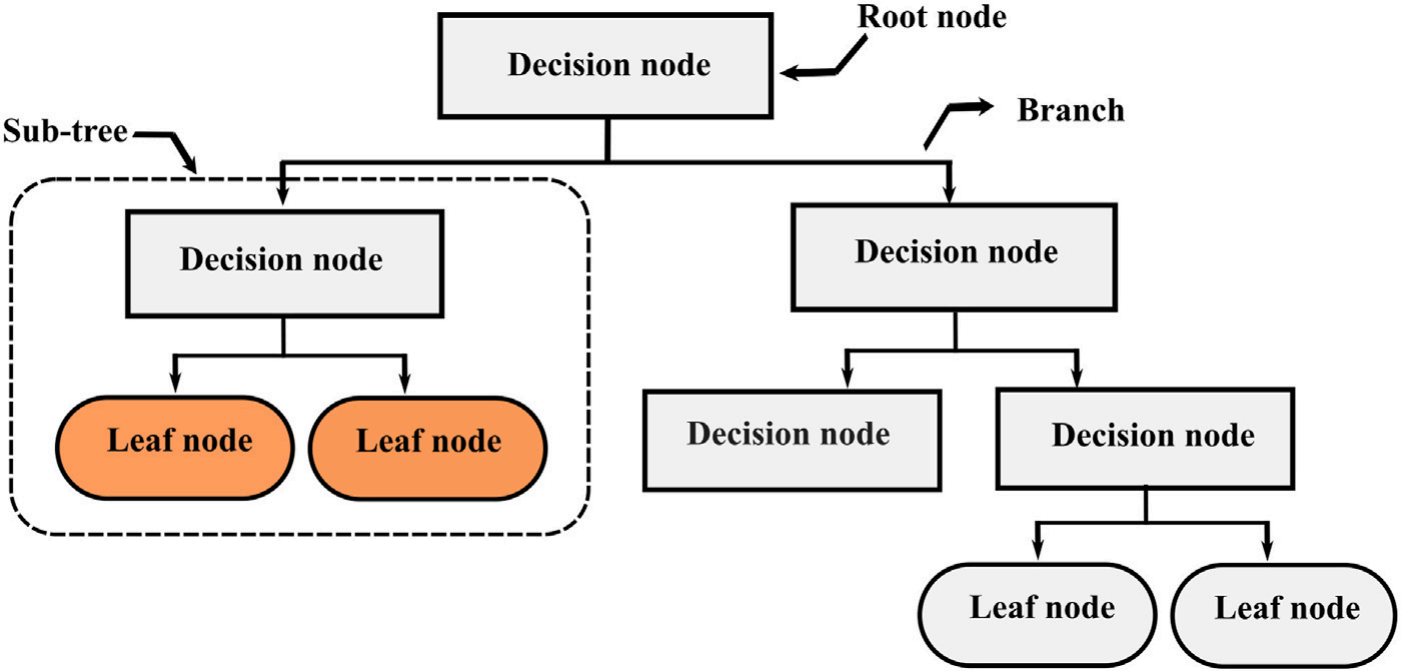
Decision trees structure.

Decision Trees (DT), also known as Classification And Regression Trees (CART) is one of the most popular supervised learning algorithms based on recursive partitioning (Jiang et al., 2020). This approach was first introduced by Breiman et al. (1984), and it relies on dividing a heterogeneous large dataset into multiple smaller homogeneous subsets, which leads to a branching structure. This structure (Figure 1) consists of nodes connected through branches. If a node does not represent an incoming edge, it is called a root. Generally, all nodes have one incoming edge and two or more outgoing edges. The nodes with no outgoing edges are called leaves. In decision trees, splitting the training data is performed by answering several questions incrementally from the topmost node to a leaf. A good question can split a heterogeneous dataset into several homogenous subsamples. Decision trees can deal with both classification and regression problems. For continuous variables, the split is performed using a threshold, the rule takes the form x<s where s is a threshold over the variable x . Contrary, when the variable is discrete, the split has the form x∈L where L is a subset of possible levels of x. When the target variable is continuous, which means we are dealing with regression, the predicted value of each subgroup is the average value of y for all observations in the training set assigned to that subgroup (Crisci et al., 2012). In contrast, when y is discrete and DT algorithm is dealing with classification problems, the most frequent level of y over the leaf observation is assigned to the target value. The basic algorithm used to build decision trees for regression matters is the Iterative Dichotomiser 3 (ID3) which uses the standard deviation reduction (SDR) to generate the decision tree. In classification situations, the ID3 algorithm uses entropy, defined as a measure of the homogeneity of subsamples, and information gain (Choudhary and Gianey, 2017). This method is widely used because of its flexibility and ease of interpretability.

### 3.4 Ensemble learning

#### 3.4.1 Bagging

Bagging, also called Bootstrap aggregating, is an ensemble method used for assembling multiple versions of a predictor to get an aggregated strong predictor (Breiman, 1996). Given a labeled training set $\left(X_{1},Y_{1}\right)\cdots \left(X_{n},Y_{n}\right)$ , bagging algorithm constructs a bootstrap replicate $\left(X_{1}^{*},Y_{1}^{*}\right)\ldots \left(X_{n}^{*},Y_{n}^{*}\right)$ , by randomly selecting samples n times with replacement from the original dataset, and then using them as new learning sets for the CART model. The final model is obtained by repeating these steps *M* times during the learning process. When predicting a numerical outcome, the aggregation algorithm averages the outcome of all predictors. If the target variable is a class label, the bagging predictor is then defined as the majority vote over the *M* models (Bühlmann, 2012). Bagging algorithms outperformed simple CART models, showing substantial gains in accuracy and significant optimization for weak learners who exhibit unstable behavior. However, bagging algorithms are sensitive to changes in training sets and can slightly reduce the performance of stable procedures (Breiman, 1996; Freund and Schapire, 1996; Bühlmann, 2012; Crisci et al., 2012).

#### 3.4.2 Random forest

Random Forest consists of a combination of tree predictors that operates as an ensemble (Breiman, 2001). These decision trees are generated by a randomized tree-building algorithm. The algorithm builds several trees using different random samples of the same size as the original training set by including certain items more than once. Additionally, at each node of the decision trees, the split considers a small random subset of features. As a result, the predictions of these trees can be different. The target value is then assigned to a certain class based on the majority vote over the prediction given by the trees (Kingsford and Salzberg, 2008). Random forests can also be used for regression, in which case the estimated value of the output variable is the average of the predictions of the trees in the forest (Choudhary and Gianey, 2017).

#### 3.4.3 Boosting

Boosting is a strategy used to enhance the accuracy of prediction models. It works by merging multiple simple models, known as weak learners, into one comprehensive and more accurate model. These weak learners, such as basic decision trees, do not have high predictive power on their own. However, when many of them are combined using a boosting algorithm, their collective accuracy significantly improves (Freund and Schapire, 1996).

The Adaboost is one of the most widely used practical boosting algorithms. The learning procedure of this algorithm starts by taking m labeled training examples $S=\left(\left(x_{1},y_{1}\right)\cdots \left(x_{m},ym\right)\right)$ , where xi belongs to some space X and it is represented as a vector of input values, and yi∈Y is the labeled output associated with xi. Boosting algorithm runs repeatedly in a series of rounds t = 1, … ,T, and every weak learner who’s given a distribution Dt, which refers to the distribution of weights assigned to the examples in the training set S at each iteration, finds a weak hypothesis ht:X→Y. The overall aim of the weak learning algorithm is to find a hypothesis, called weak hypothesis, that minimizes the weighted error t associated to Dt. The final outcome of the boosting algorithm is a combination of all the weak hypotheses, where each one is assigned a weight (αt) according to its importance. The more accurate a weak hypothesis is, the higher its weight. This final combination is a kind of “majority vote” of all the weak hypotheses, and it is much more accurate than any of the individual weak learners. Mathematically, the final hypothesis H is represented as a weighted majority vote of the weak hypotheses, where every hypothesis ht is multiplied by a weight αt (Freund and Schapire, 1996). Boosting is effective at reducing both random variability (variance) and systematic error (bias) in the predictions. It also has a unique feature where it focuses more on the more challenging examples, based on the performance of the previous weak learners. This makes boosting algorithms perform better than other methods like bagging, and makes them less sensitive to changes in the training data (Freund and Schapire, 1996).

### 3.5 Kernel-based algorithms

#### 3.5.1 Reproducing kernel Hilbert spaces (RKHS)

Reproducing kernel Hilbert (RKHS) is a semi-parametric regression model applied for the first time on marker genotypes by Gianola et al. (2011). This method has shown great computational potential, especially when *p* >> *n.* RKHS is a Hilbert space (H) of functions where every function can be thought of as a point in Euclidean space, and is assumed to be bounded and linear. In other words, if two functions f and g have close norms $\left\|f\left(\varkappa \right)-g\left(x\right)\to 0\right\|$ , they also have close values $\left|f\left(x\right)-g\left(x\right)\to 0\right|$ . The learning task of RKHS can be described as follows: Let $x_{i}$ be a vector of marker genotypes (input), $y_{i}$ a vector of genetic values (output), and $g\left(x\right)$ an unknown function of genetic effects.

To infer g , RKHS proceeds by defining a space of functions from which an element $\hat{g}$ will be chosen if it minimizes the loss function bellow:

$$l\left(g|\lambda \right)={\left\|y-g\right\|}^{2}+\lambda {\left\|g\right\|}_{H}^{2}$$ (5)

Where λ is a regularization parameter that controls tradeoffs between goodness of fit and model complexity, H represents a Hilbert space, and ${\left\|g\right\|}_{H}^{2}$ is the square of the norm of g on H. The square of the norm measures the model complexity. According to Manton and Amblard (2014), RKHS theory can be used to solve three types of problems:

(i) when the problem is defined over a subspace that happens to be RKHS. This suggests that mapping the problem space into a higher dimensional space makes the problem easier. Genomic selection poses a high-dimensional challenge as the number of genotypes (p) typically exceeds the number of individuals (n). By leveraging an RKHS framework, it becomes possible to mitigate this dimensionality and facilitate solving such problems. Introducing a Gaussian kernel allows for transforming the genotypic data into an appropriate RKHS representation, whereby subsequent linear regression models can be effectively used for predicting genetic values within this reduced-dimensional space.
(ii) when a problem has a positive semi-definite function: In the field of genomic selection, a critical component is the genetic relationship matrix (also referred to as the kinship matrix), which quantifies the genetic similarity between individuals. This function serves an important purpose in correcting for confounding factors such as population structure and familial relatedness in association studies. Utilizing a reproducing kernel Hilbert space is one solution to the problem that high-dimensional genotypes present. By applying this approach, we can leverage the kernel trick to effectively handle and make more manageable this complex problem.
(iii) When the data points can be embedded into a RKHS with the kernel function capturing the characteristics of the distance function, given all the data points and a function determining the distance between them Nayeri et al. (2019). One common task in genomic selection is to group individuals based on their genotypes. This is typically done for purposes such as identifying subpopulations or accounting for population structure. To achieve this, the genotypes can be embedded into a reproducible Kernel Hilbert Space using an appropriate kernel function, such as a Gaussian or linear kernel. By doing so, we are able to capture the genetic similarity among individuals. The clustering algorithm operates within this RKHS and aims to find clusters that are well-separated in the RKHS even if they may not appear well-separated in the original genotype space.

#### 3.5.2 Support vector machines

Support vector machines (SVM) is a non-parametric algorithm proposed by Cortes and Vapnik (1995). It was first conceived for two-group classification problems; however, it is widely used nowadays for both regression and classification. When dealing with clustering, the aim of SVM algorithm is to identify an optimal hyperplane defined as a boundary that maximally separates classes (Jiang et al., 2020). When data points are linearly separable, the SVM algorithm performs a linear classification and the optimal hyperplane is found using numerical optimization (Crisci et al., 2012). Otherwise, SVM can perform a non-linear classification using the Kernel function. Gaussian kernel function is used to map the data points from a data space to a high-dimensional feature space. In the feature space, small spheres appear to enclose the image of data, these spheres are mapped back to the data space and form cluster boundaries that enclose data points of the same cluster (Ben-Hur et al., 2001). The boundaries should maximize the margin between them and the classes to minimize the classification error (Mahesh, 2020). When the SVM algorithm is applied to regression problems, the loss function should include a distance measure. The possible loss functions are the quadratic, Laplacian loss function, Huber and the insensitive loss function (Gunn, 1998). SVM algorithms can result in highly accurate predictions due to their flexibility. However, they’re described as a black box because no metrics are provided for how predictors optimize the hyperplane, which makes the predictions hard to interpret (Jiang et al., 2020).

### 3.6 Nearest neighbors

Nearest neighbors model is one of the most simple and intuitive machine learning algorithms. The idea of this approach is to forecast the value of a target variable $y_{i}$ associated with an input variable $x_{i}$ based on the distance between $x_{i}$ and other data points. Generally, Euclidean distance is used, but there are other methods to calculate this distance, such as Manhattan distance (Zhang, 2016). In classification, $y_{i}$ is assigned to the class label of the majority of the nearest data points in the space. Alternatively, when dealing with regression, the predictor is the average of the output over the nearest neighbors (Crisci et al., 2012). The K-nearest neighbors (KNN) is the most popular algorithm in this category. It is based on the same idea that the nearest patterns to a datapoint $x_{i}$ deliver useful label information. The unknown parameter K decides how many neighbors will be considered in the learning process (Kramer, 2013). The number of neighbors K has a significant impact on the performance of the algorithm. An optimal K is the one that strikes a balance between overfitting (low bias but high variance) and underfitting (low variance but high bias). Some authors suggest K to the square root of the number of observations in the training set (Zhang, 2016).

### 3.7 Deep neural networks

**FIGURE 2:**
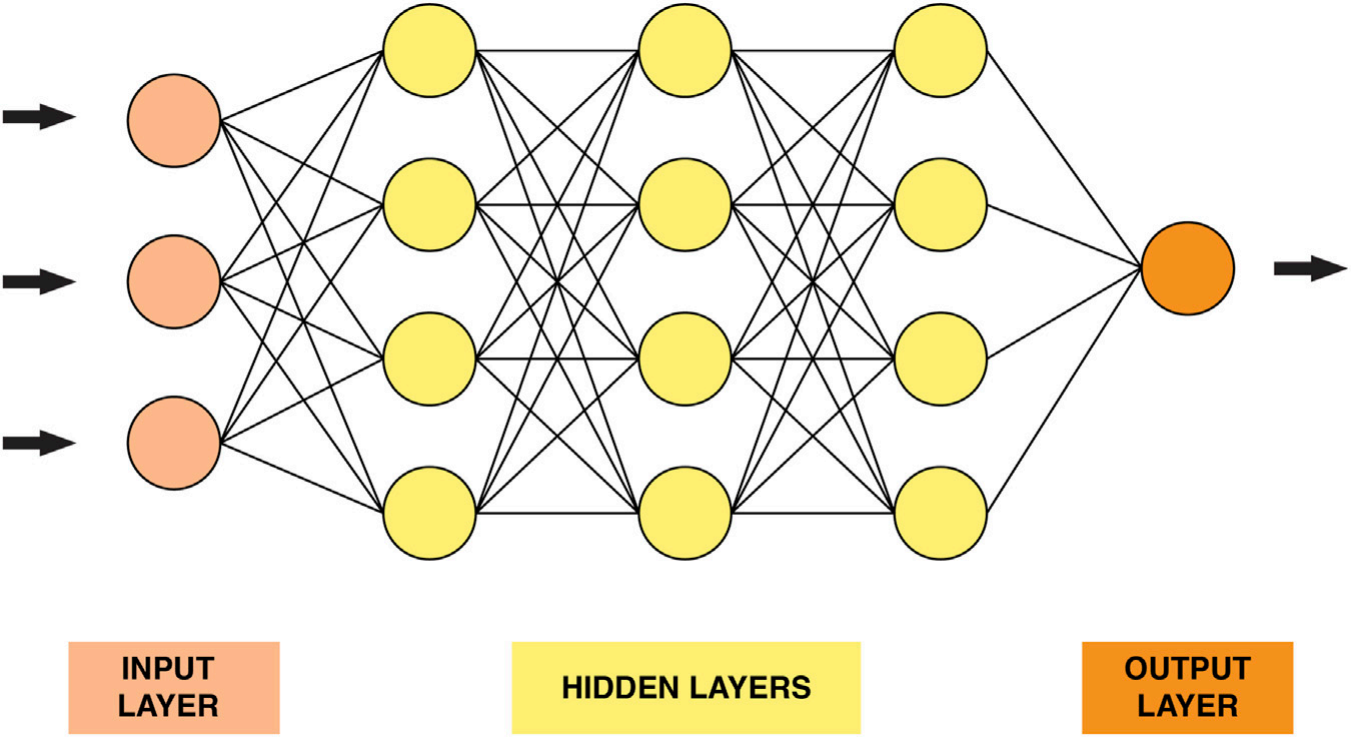
A graphical representation of a simple neural network.

Deep learning is a family of powerful learning methods capable of recognizing complex patterns in raw data (Vieira et al., 2020). The well-known Rosenblatt “perceptron” proposed in the 1950s was the first attempt to conceive a model closely analogous to the perceptual processes of the human brain (Rosenblatt, 1957). Deep neural networks’ (DNN) structure (Figure 2) consists of stacked layers of connected neurons. In other words, the DNN model comprises a certain number of layers, each layer contains several neurons. Each neuron is connected to the neurons in adjacent layers through weights that reflect the strength and direction of the connection (excitatory or inhibitory) (Montesinos-López et al., 2021). DNN models are characterized by their depth, size and width. The number of layers that a DNN contains, excluding the input layer, is called depth. The total number of neurons in the model is referred to as the size. Finally, the width of the DNN is the layer that comprises the largest number of neurons.

When running DNN, a set of observations X enter the model through the input layer. The observations $x_{i}$ are the input and the output of this layer. In the hidden layers of the DNN, every neuron of a given layer receives from the layer of lower hierarchical level, the weighted sum of its neurons’ output, and then passes it through an activation function to drive it as an output for that neuron. In the hidden layers, the most widely used activation functions are the rectified linear unit, hyperbolic tangent activation and the sigmoid function. In the output layer, the DNN is meant to perform either a classification or a regression based on the nature of the target variable. When dealing with classification, the number of neurons in the output layer is equal to the number of classes. Additionally, different activation functions could be used according to the type of the target variable. Softmax is used for categorical variables, the exponential function for count data and the sigmoid function for binary outcomes (Vieira et al., 2020; Montesinos-López et al., 2021). In regression problems, the output layer represents the estimated values of the target variables and linear activation functions are applied. The most successful activation function when dealing with a continuous variable is the rectified linear unit (ReLU) (Bircanoğlu and Arıca, 2018). The tanh activation function is used in DNN to introduce non-linearity in the model and to allow the model to learn from both positive and negative weights since it is centered around zero (unlike the sigmoid function). It is typically used in the hidden layers.

Like other ML models, training DNN consists of choosing optimal weights that minimize the differences between real and estimated values of the target variable. The gradient descent is used to minimize the loss function. These parameters need to be updated during the learning process. When first training the DNN model, the weights are randomly initialized. Once an observation has entered the model, the information is forward propagated through the network until it predicts a certain output value. The gradients of the loss function are then computed using a hyperparameter called the learning rate η, which indicates how big the steps of gradient descent should be, and then used to update the function parameters (weights and biases). Backpropagation is another efficient method of computing gradients. The concept of this method is based on the fact that the contribution of each neuron to the loss function is proportional to the weight of its connection with the neurons of the following layer. Therefore, these contributions could be calculated starting from the output layer and backpropagated through the network using the weights and the derivative of the activation function (Pereira and Borysov, 2019; Vieira et al., 2020; Montesinos-López et al., 2021).

Deep learning comprises a wide variety of architectures. The most popular ones are the feedforward networks, also called the multilayer perceptron (MLP), recurrent neural networks (RNN) and the convolutional neural networks (CNN).

#### 3.7.1 Multilayer perceptron (MLP)

The multilayer perceptron (MLP) is a layered feedforward network where all layers are fully connected. Every neuron of a given layer is connected to neurons of the adjacent layer, the information flows in a single direction. In other words, there are no intralayer or supralayer connections. MLPs are found to be powerful and simple to train. However, these networks are not suitable to deal with spatial or temporal datasets and they’re prone to overfitting (Montesinos-López et al., 2021).

#### 3.7.2 Recurrent neural networks (RNN)

In Recurrent Neural Networks (RNN), information flows in both directions. Every neuron has three types of connections: incoming connections from the previous layer, ongoing connections toward the subsequent layer, and recurrent connections between neurons of the same layer (Montesinos-López et al., 2021). This recursive structure allows this network to have some notion of memory since the output of a layer depends on both current and previous inputs. RNN are frequently used to model space-temporal structures. It is also used in the fields of natural language processing and speech recognition (Pereira and Borysov, 2019; Zingaretti et al., 2020).

#### 3.7.3 Convolutional neural networks (CNN)

Convolutional Neural Networks (CNN) are designed to accommodate situations where data is represented in the form of multiple arrays. The input variable can have one-dimension such as SNPs, two dimensions such as color images, or three dimensions for videos or volumetric images (LeCun et al., 2015). The architecture of CNNs is made up of convolutional and pooling layers followed by fully connected neural networks (Pereira and Borysov, 2019). When training CNNs, the first two types of layers, namely, convolutional and pooling layers, perform feature extraction. The fully connected neural network is meant to perform the classification or the regression task. In the convolutional layer, a mathematical operation is performed to generate one filtered version of the original matrices of the input data. This convolutional operation is called “kernel” or “filter”. A non-linear activation function, generally ReLU, is applied after every convolution to produce the output, which is organized as feature maps. The pooling operation comes after to smooth out the results, its role is to merge semantically similar features into one. In other words, pooling reduces the number of parameters and makes the network less computationally expensive. Max pooling is a typical pooling operation that proceeds by extracting patches from the feature maps, determining the maximum value in each patch, and then eliminating all the other values. Finally, after turning the input matrices into a one-dimensional vector, the features are mapped by a network of fully connected layers similar to the aforementioned feedforward deep network to obtain the final output, the probabilities of a given feature belonging to a given class for example,. The output of the fully connected neural network is fed to another different activation function to perform classification or regression based on the output variable (Yamashita et al., 2018). CNNs have been successfully applied in visual and speech recognition, natural language processing, and various classification tasks (LeCun et al., 2015; Yamashita et al., 2018; Pereira and Borysov, 2019).

## 4 Performance fitness and error metrics

Machine learning algorithms need to be rigorously evaluated in order to confirm their validity in understanding complex datasets and hence extend the use of this model in different datasets. Generally, the performance of ML models is assessed using Performance Fitness and Error Metrics (PFEMs), defined as mathematical constructs used to measure how close the predicted and real observed values of a given variable are. Choosing the right metric for assessing the performance of a predictor is very delicate because a limited understanding of the behavior of algorithms can lead to misinterpretations of results and thus false assumptions. In addition, PFEMs are used differently when dealing with regression and classification problems.

In regression, performance metrics are based on calculating the distance between predicted and real values using subtraction or division operations, sometimes supplemented with absoluteness or squareness. Moreover, PFEMs in regression also investigate the distribution of residuals, whether it is random or regular, which indicates that the regression model does not explain all the regularity in the dataset. The most common PFEMs used in regression are (Table 1): mean square error (MSE) or root mean square error (RMSE), normalized mean squared error (NMSE), correlation coefficient (R), r squared (R^2^), mean absolute error (MAE), and mean absolute percentage error (MAPE). They are easy to interpret, straightforward, and they indicate the magnitude of the difference between measured and predicted values (Naser and Alavi, 2021). The interpretation of these metrics can be found elsewhere (Botchkarev, 2018).

**TABLE 1:** Common performance metrics used for the evaluation of regression models.

Metric abbreviation	Metric name	Metric formula
MSE	Mean squared error	$MSE=\frac{1}{N}\sum_{n=1}^{N}{\left[y\left(n\right)-\hat{y}\left(n\right)\right]}^{2}$
RMSE	Root mean squared error	$RMSE=\sqrt{MSE}$
NMSE	Normalized mean squared error	$NMSE=\frac{\sum_{n=1}^{N}{\left[y\left(n\right)-\hat{y}\left(n\right)\right]}^{2}}{\sum_{n=1}^{N}{\left[y\left(n\right)-\underline{y}\right]}^{2}}$
MAE	Mean absolute error	$MAE=\frac{1}{N}\sum_{n=1}^{N}\left|{\left[y\left(n\right)-\hat{y}\left(n\right)\right]}^{2}\right|$
MAPE	Mean absolute percentage error	$MAPE=\frac{100}{N}\sum_{n=1}^{N}\left|\frac{{\left[y\left(n\right)-\hat{y}\left(n\right)\right]}^{2}}{y\left(n\right)}\right|$
$R^{2}$	Coefficient of determination	$R^{2}=1-NMSE$

Where N (1 , ..., n ) is the number of observations, $y\left(n\right)$ refers to observed values, and $\hat{y}\left(n\right)$ refers to the estimated values.

**FIGURE 3:**
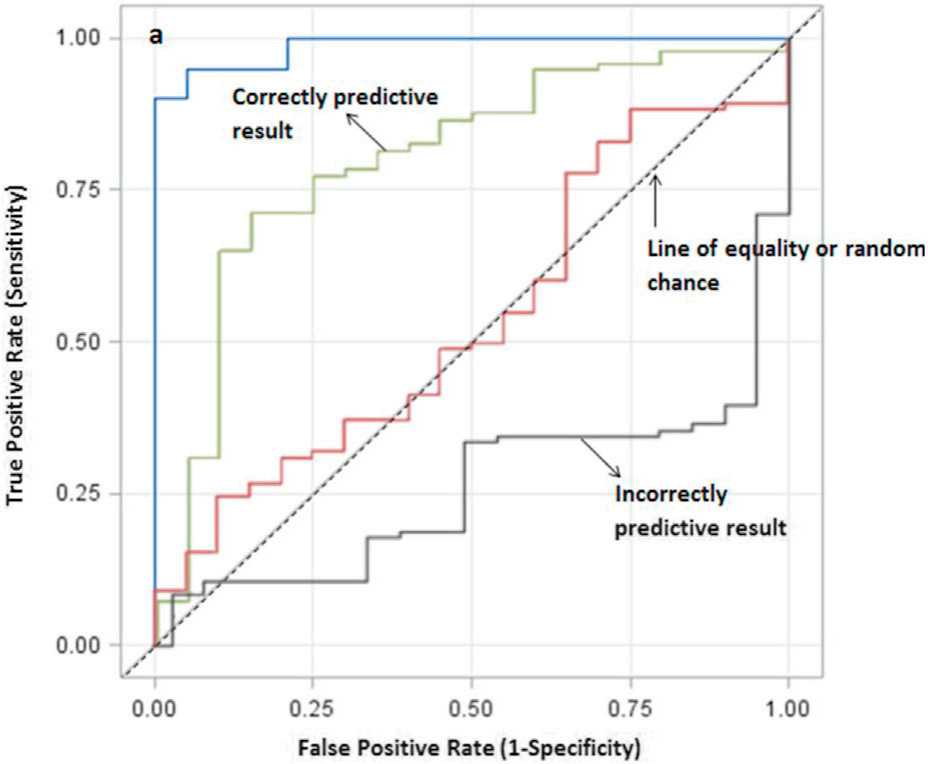
Interpretation of ROC curves of varying sensitivity and specificity. The sensitivity and the specificity of the test increases as the curve approaches the point a (x = 0, y = 1). The closer the curves are to the diagonal line the less precise they are. From “ROC-ing along: Evaluation and interpretation of receiver operating characteristic curves” by Carter et al. (2016).

Classification models are meant to categorize data into distinct classes. Therefore, assessing the performance of classifiers relies on a confusion matrix where columns represent the predicted values, while rows represent the actual values as described in Figure 3, where *TP* refers to true positives, *TN* denotes true negatives, *FP* denotes false positives, and *FN* refers to false negatives. The performance of classifiers is often evaluated using prediction accuracy (PAC), sensitivity or recall, specificity, and precision. Based on the confusion matrix, these metrics are defined as below:

$$PAC=\frac{TP+TN}{TP+FP+TN+FN},precision=\frac{TP}{TP+FP},recall=\frac{TP}{TP+FN},specificity=\frac{TN}{TN+FP}$$ (6)

Other methods based on the aforementioned metrics have also been broadly used in assessing the performance of classifiers. The F1 score that combines both precision and recall in a harmonic mean in the following formula:

$$F1\;score=2\;x\;\frac{precision\;x\;recall}{precision+recall}$$ (7)

Moreover, Matthews (1975) introduced a coefficient used to measure the performance of binary classifiers, called the Matthews correlation coefficient (MCC). This coefficient combines all four measures in the confusion matrix, and thus it is qualified as the most informative metric especially when a significant imbalance in class sizes is noticed (Nayeri et al., 2019). MCC formula is represented below:

$$MCC=\frac{TP\;x\;TN-FP\;x\;FN}{\sqrt{\left(TP+FP\right)\;x\;\left(TP+FN\right)\;x\;\left(TN+FP\right)\;x\;\left(TN+FN\right)}}$$ (8)

**FIGURE 4:**
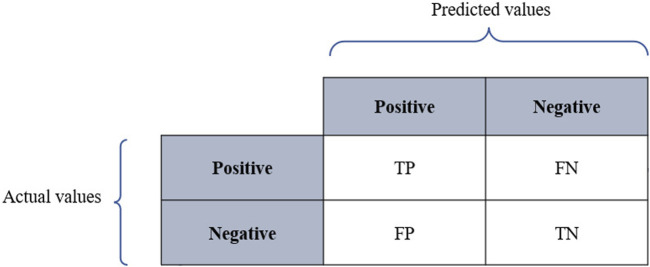
Confusion matrix.

Another criterion widely used to measure the performance of classifiers is the Area Under the Receiver Operating Characteristic (ROC) curve (AUC). The ROC curve visualizes the tradeoff between sensitivity and specificity. In other words, the curve captures the ratio of false to true positive rates under variation of the decision threshold (Hoffmann et al., 2019). Generally, good performance is detected when the curve is high and close to the left in the ROC space. In contrast, an inaccurate method has a curve close to the main diagonal (Figure 4). Thus, when comparing several ML models, the one with the highest AUC value is the most accurate (Metz, 1978).

## 5 Machine learning models applied to genomic prediction in animal breeding

Machine learning algorithms have been widely used in various fields. Their ability to discover patterns in large, messy datasets has driven researchers to investigate their performance in dealing with complex models and nonlinearities in large datasets. Animal breeding in the post-genomic era is a domain that deals with high-dimensional marker datasets such as genomics, epigenomics, transcriptomics, proteomics and metabolomics. The most commonly used marker data sets in animal breeding are single nucleotide polymorphism (SNPs) data sets that represent the genetic variation in a genome. SNP markers data sets are very large, for example, the data set resulting from genotyping 2,000 individuals for 10,000 SNP markers, contains 20 million data points. Furthermore, they can be complex and noisy due to genotyping errors, missing data, batch effects, and biological variability. Copy number variation (CNV) is another valuable form of genetic variation that complements SNPs analysis. CNV datasets are used to investigate diversity within populations (Yang et al., 2018). They can serve as informative markers for marker-assisted selection by identifying CNVs associated with desirable traits (Ma et al., 2018), and genomic prediction to enhance the accuracy of predicting breeding values (Hay et al., 2018), etc. In addition, microarray data provide valuable information concerning gene expression, by measuring the mRNA expression levels of tens of thousands of genes. Gene expression datasets are known to be massive (large number of genes) and redundant, and thus, their manipulation requires a lot of pre-processing and dimensionality reduction (Liu and Motoda, 2007). Applying machine learning models is hence becoming attractive in genomics, due to their potential in dealing with large, noisy data and modeling minor nonadditive effects as well as interactions between phenotypes and genotypes.

Machine learning models have several important applications in genomics. Through the introduction of sophisticated algorithms and computational models, ML can be trained using large datasets of genotypes and phenotypes to predict animals’ breeding values for certain traits. This would enable an accurate selection of animals with the highest genetic merit and allow for more informed breeding decisions. ML models have successfully been implemented to predict genomic breeding values across various animal species, including dairy cattle (Beskorovajni et al., 2022), beef cattle (Srivastava et al., 2021), pigs (Zhao et al., 2020), and broilers (González-Recio et al., 2008). The estimated GEBVs provide an accurate prediction of animals’ genetic potential and thus identify animals with high genetic potential that surpass the population average. Therefore, ML models can have a valuable role in allowing breeders to make more precise breeding decisions, leading to faster genetic progress.

In addition, machine learning algorithms can also be deployed to predict disease occurrence based on integrated information of genotypes and health records. For example, Ehret et al. (2015) applied ML to encounter a serious health problem in the intensive dairy industry, which is subclinical ketosis risk. The authors proposed an ANN to investigate the utility of combining metabolic, genomic and milk performance in predicting milk levels of β-hydroxybutyrate. Data comprised SNP markers, and weekly records of the concentrations of glycerophosphocholine, phosphocholine, and milk composition data (milk yield, fat and protein percentage). The deep learning model deployed provided an average correlation between real and predicted values up to 0.643 when incorporating information about metabolite concentration, milk yield, and genomic information.

Moreover, ML models can be coupled with GWAS and population genomics to identify genetic variants and biological pathways linked to specific phenotypic traits. A deep learning framework was proposed by Zeng et al. (2021) to predict quantitative phenotypes of interest and discover genomic markers considering the zygosity of SNP information from plants and animals as input. Furthermore, ML models can be used to impute moderate-density genotypes when genotyping large populations can be expensive and time-consuming. ML models can accurately infer missing genotypes and fill the gaps to create moderate density genotypes. This has already been implemented in the beef cattle genomic dataset (Sun et al., 2012).

Taken together, ML models appear to be a powerful tool for enabling more accurate predictions, targeted selection, and an improved understanding of genetic mechanisms. However, when training ML models on biological data, several challenges can occur. For example, when using markers data, environmental data, and phenotypic records all together to predict a certain variable, the large heterogeneity of the input data can be a hurdle. Therefore, it is indispensable to perform a pre-processing step that includes formatting, cleaning, scaling, and normalizing the data. This step ensures that the data is prepared to optimize the performance and accuracy of the machine learning model. Markers data sets are usually massive and comprise a lot of noise. Using the raw data can lead to a low performance and overfitting. Thus, performing feature selection is vital when manipulating omics data in order to reduce the dimensionality of the data by selecting relevant features while eliminating noise from the model. Multiple methods can be used to perform feature selection including statistical methods, correlations, or hypothesis testing. Recently, ML models were proved to be very powerful in feature selection. The most broadly used machine learning-based methods for feature selection are filters, wrappers, and embedded methods that combine filter and wrapper methods (Tadist et al., 2019). Machine learning-based feature selection is widely used when manipulating animal species marker data sets. Finally, when training ML models on biological data, several steps should be performed to ensure the quality of the data fed to the model. In addition, adjusting the hyperparameters and generalizing the model through regularization techniques are also central to optimizing the performance of the model. There are multiple techniques to optimize ML models, such as gradient descent, stochastic gradient descent, random search, grid search, Bayesian optimization, and genetic algorithms.

Now that we have discussed the overall applications of ML models in genomic prediction and the multiple issues encountered while implementing those models on markers data, we will review, in this section, some of the published studies on the application of different ML models for genomic prediction in animal breeding, feature selection, and genotype imputation separately, to provide a meta-picture of their potential in terms of prediction accuracy and computational time. Data sets and different machine learning models applied to genomic prediction in a handful of the reviewed papers are summarized in Table 2. In Supplementary Materials; Table 1 contains the full summary of the reviewed papers, and Table 2 presents the programming languages and packages used to train the models in the aforementioned studies.

**TABLE 2:** Machine learning models applied to genomic prediction in animal breeding.

Year	Authors	Species	Breed	No. of individuals	No. of markers	Response variable	ML algorithms	Aim of the study
2016	Naderi et al.	Dairy cattle (simulated)	-	20000 females and 400 males	50025 and 10005 SNPs	Subclinical Ketosis	ANN (MLP)	Building an ANN for an earlier prediction of subclinical Ketosis in lactation
2016	Yao et al.	Dairy cattle	Holstein	3000 genotyped 792 genotyped and phenotyped	57491 SNPs	RFI	SVM (semi-supervised learning)	Describing a SVM-based semi-supervised learning model, and applying it for genomic prediction of residual feed intake
2018	Li et al.	Beef cattle	Brahman	2093	40184 SNPs	BW	RF, GBM, XGBoost	Assessing the efficiency of three ML methods in identifying the top-ranked SNPs and using the subsets of SNPs to construct genomic relationship matrices for estimating genomic breeding values
2020	Liang et al.	Beef cattle	Simmental	1217	671900 SNPs	CW, LW, EMA	Adaboost.RT (integrated SVR), KRR, RF	Applying ensemble learning models to predict genomic breeding values of three economic traits
2020	Abdollahi-Arpanahi et al.	Dairy cattle	Holstein	1170	57749 SNPs	SCR	MLP, CNN, RF, GB	Comparing the predictive performance of two deep learning methods, two ensemble learning methods, gradient boosting and two parametric methods (GBLUP and Bayes B)
		Simulated data	-	-	100 and 1000 QTNs	A quantitative trait		
2021	Chen et al.	Beef cattle	Nellore	18	16,423 genes	FE	RF, XGBoost, RX, SVM	Applying Rf, XGBoost and RX to identify small subsets of biologically important genes to classify animals into High Feed Efficiency and Low Feed Efficiency
2021	Srivastava et al.	Beef cattle	Hanwoo	7324	53866 SNPs	CWT, MS, BFT EMA	RF, XGB, SVM	Comparing the predictive ability of three ML models in predicting phenotypes from genotypes
2021	Wang et al.	Pig	Yorkshire	2566	44922 SNPs	TNB, NBA	SVR, KRR, RF, Adaboost.R2	Exploring and comparing the prediction ability of fourML models to GBLUP, ssGBLUP and bayesian methods in genomic prediction of reproductive traits
2021	Beskorovajni et al.	Dairy cattle	Holstein	92	-	MFP, MPP, CM, FM, LIV, SCE, HCR, CCR, DSB, SSB, GL	MLP	Predicting yield and fertility traits using an MLP model based on the Broyden-Fletcher-Goldfarb-Shanno iterative optimization algorithm for genomic selection
2021	An et al.	Beef cattle	Simmental	1301	671990 SNPs	Cosine Kernel based KRR (KcRR),SVR	LW, CW, EMA	Assessing the prediction accuracies of 12 traits with various heritabilities and genetic architectures using parametric methods (GBLUP and Bayes B), and two machine learning models (KcRR and SVR)
		Dairy cattle	Holstein	5024	42551 SNPs		MY, MFP, SCS	
		Pig	-	3534	43494, 43407, and 43412 SNPs for each trait		T1, T2, T3	
		Simulated data	-	4000	50 SNPs for each trait (3 traits)		T1, T2,T3	

A summary of a handful of the reviewed researches in the paper. For the full version of the table please view Supplementary Materials.

### 5.1 Genomic prediction

The wide majority of traits of interest in animal breeding are presumed to be influenced by many genomic regions with complex interactions. Kernel-based methods are gaining consideration over conventional regression models due to their capacity to capture non-additive effects. A more succinct description of kernel-based methods applied to GP can be found in Morota and Gianola (2014). González-Recio et al. (2008) used the F-metric model, kernel regression, reproducing kernel Hilbert spaces (RKHS) regression, and Bayesian regression to predict mortality in broilers and see how well they did compared to the standard genetic evaluation (E-BLUP), which is only based on pedigree information. The dataset contained records for mortality rates for 12167 progeny of 200 sires with a total of 5523 SNPs. The authors concluded that kernel regression and RKHS regression had a low residual sum of squares and increased the accuracy from 25% to 150% relative to other methods, and thus the authors recommended their utility in the genomic prediction of early mortality in broilers. An et al. (2021) developed another kernel-based algorithm named Cosine Kernel-based Ridge Regression (KcRR) to perform genomic prediction using simulated and real datasets. The simulated dataset included 4000 individuals and concerned three quantitative traits with various heritabilities (0.36, 0.35, and 0.52). Meanwhile, the real data concerned three species: a Chinese Simmental beef cattle dataset contained 1,301 bulls, with a total of 671990 SNPs and concerned three traits of interest: live weight (LW, kg), cold carcass weight (CW, kg), and eye muscle area (EMA, cm^2^). The pig dataset included 3,534 animals, and finally, the German Holstein cattle dataset included 5,024 bulls with a total of 42551 SNPs that concerned three phenotype traits, milk yield (MY, kg), milk fat percentage (MFP,%), and somatic cell score (SCS). The designed model consisted of a kernel-based ridge regression, which is a ridge regression built in a higher dimensional feature space that uses a Cosine similarity matrix (CS matrix) instead of the genomic relationship matrix (G matrix). The difference between these two matrices is that the CS matrix measures the cosine of the angle between two projected vectors, and the G matrix in an m-dimensional feature space where m is the number of SNP markers. For comparison purposes, a 20-fold cross-validation approach was used to evaluate the prediction accuracy of KcRR to that of GBLUP, BayesB, and SVR. The authors have also simulated for the quantitative traits different heritabilities, and genetic architectures, including one major gene and a large number of genes with minor effects, a number of genes with moderate effects and many genes with small effects, and finally a large number of genes with small effects, in order to assess the performance and consistency of these methods. Overall, KcRR had the best prediction accuracy among the methods, in addition, it performed stably for all traits and genetic architectures, which confirms its reliability and robustness. Therefore, An et al. (2021) suggested the use of KcRR and the CS matrix as a potential alternative in future GP. Zhao et al. (2020) investigated the performance of SVM in a pig dataset containing 3,534 samples with a different number of SNPs for each trait respectively 45,025, 45,441, 44,190, 44,151, and 44,037 SNPs for T1, T2, T3, T4, and T5. For training the SVM model, a suitable kernel function was selected. The authors tested the prediction ability of four commonly used kernel functions namely, the Radial Basis Function (RBF), the Polynomial Kernel Function, the Linear Kernel Function, and the Sigmoid Kernel Function in previously published pig and maize datasets. The findings demonstrated that SVM-RBF had the best performance, the SVM-sigmoid and the SVM-poly models had similar accuracies, and the SVM-linear had the lowest accuracy. As a result, the authors chose using the SVM-RBF model to adjust the hyperparameters of the final SVM model. Afterwards, the authors evaluated the performance of SVM-RBF, GBLUP and BayesR in fitting the five pig datasets, using a 10-fold cross-validation approach. Overall, the performance of the trained models was similar. However, the SVM model performed better than BayesR but worse than GBLUP in terms of time, and better than GBLUP but worse than BayesR in terms of memory.

Ensemble learning has been broadly used in the genomic prediction of animal breeding values. Naderi et al. (2016) studied the use of RF for genomic prediction of binary disease traits using simulated data from 20,000 cows with different disease incidence scenarios, different heritability (h2 = 0.30 and h2 = 0.10), and different genomic architecture (725 and 290 QTL, populations with high and low levels of linkage disequilibrium). The training set contained 16,000 healthy cows, and the testing data contained the remaining 4,000 sick cows. Afterwards, the number of sick cows was increased progressively by moving 10% of the sick individuals to the training data, ensuring that the size of both the training and testing data remained constant. This study compared the performance of RF and GBLUP using the correlations between estimated genomic breeding values and true breeding values, and the area under the curve (AUROC). The results confirmed that RF had a great advantage in the binary classification for scenarios with a larger marker density. In addition, the best prediction accuracies of RF (0.53) and GBLUP (0.51), and the highest values of AUROC for RF (0.66) and for GBLUP (0.64), were achieved using 50,025 SNPs, a heritability of 0.30, 725 QTL, and a disease incidence similar to the population disease incidence (0.20). The authors also noted that the genetic makeup of the population had an impact on the performance of RF and GBLUP. However, the variability was more pronounced for RF than for GBLUP.

A boosting algorithm called L2-Boosting was suggested by González-Recio et al. (2010) to forecast the progeny test predicted transmitting abilities for the length of productive life (PL) in a dairy cattle dataset, and the average food conversion rate records in a broiler dataset. The dairy cattle data set consisted of 4702 Holstein sires with a total of 32611 SNPs, and the broiler dataset comprised 394 sires of a commercial broiler line with 3,481 SNPs. The L2-Boosting algorithm proceeds by combining two weak learners, namely, ordinary least squares (OLS) and non-parametric (NP) regression. The performance of OLS-Boosting and NP-Boosting was compared to Bayesian LASSO (BL) and Bayes A regression. The results showed that OLS-Boosting had the lowest bias and mean-squared errors (MSEs) in both the dairy cattle (0.08 and 1.08, respectively) and the broiler (0.011 and 0.006, respectively) data sets. The authors concluded that L2-Boosting with a suitable learner represents a good alternative for genomic prediction, providing high accuracy and low bias in a short computational time.

In another study, a bagging approach using GBLUP (BGBLUP) was performed to predict the genomic predicted transmitting ability (GPTA) of young Holstein bulls for three traits: protein yield (PY), somatic cell score (SCS), and daughter pregnancy rate (DPR) (Mikshowsky et al., 2017). The dataset consisted of 17276 Holstein bulls with a total of 57169 SNP markers, and it was split into a reference population set used to train the model and a testing set for the evaluation. The aim of the proposed bagging approach was to create 50 bootstraps containing bulls selected randomly, with replacement, from the reference population, until each bootstrap reaches the same number of individuals as the original reference population. GBLUP was applied to predict the GEBVs of individuals for each trait. According to the results, GBLUP outperformed BGBLUP in the genomic prediction for PY, SCS, and DPR, the correlations between the real and predicted values of each trait for GBLUP were 0.690, 0.609, and 0.557, and 0.665, 0.584, and 0.499 for BGBLUP. In summary, the authors found no advantage to using BGBLUP over GBLUP for genomic prediction.

For comparison purposes, several studies have deployed various machine learning methods to forecast and compare their predictive accuracies when trained using genomic data. For example, Ogutu et al. (2011) compared the performance of three machine learning models, namely RF, stochastic gradient boosting, and SVMs, in estimating genomic breeding values. A simulated dataset of 2326 genotyped and phenotyped individuals and 900 individuals who lacked phenotypic records was used. As a performance metric, Pearson correlations were used between the simulated values and the predicted values from the validation set, as well as between the predicted and real breeding values for non-phenotyped individuals. The results showed that stochastic gradient boosting and SVM had better correlations between the simulated values and predicted values compared to RF. However, RF provided reasonable rankings of the SNPs, which can be useful for identifying markers for further testing. In conclusion, stochastic gradient boosting and SVM are found to be able to accommodate complex relationships and interactions in marker data such as epistasis. They have also outperformed RF in the genomic prediction of the quantitative trait, however, SVM was computationally intensive due to the grid search for tuning the hyper-parameters. In contrast, Srivastava et al. (2021) found different conclusions when evaluating the performance of RF, XGB, and SVM in predicting four traits namely, carcass weight (CWT), marbling score (MS), backfat thickness (BFT) and eye muscle area (EMA) of 7234 Hanwoo cattle. According to this study, XGB yielded higher correlations for CWT, MS, (0.43, 0.44, respectively) compared to GBLUP (0.41, 0.42), and lower (0.23, and 0.31) than GBLUP (0.35, and 0.38) for BFT, and EMA. Meanwhile, GBLUP delivered the lowest MSE for all traits. Among the ML methods, XGB had the lowest MSE for CWT and MS, and SVM provided the lowest MSE for BFT and EMA. Despite the good performance of XGB and SVM, the authors still concluded that there was no advantage to using ML methods over GBLUP.

Liang et al. (2021), compared the performance of Adaboost.RT, SVR, KRR, RF to the conventional GBLUP in predicting breeding values for cattle growth traits in Chinese Simmental cattle (carcass weight, live weight, and eye muscle area), using a dataset of 1,217 young bulls with a total of 671990 SNPs. Contrary to the previous study, the authors recommended using ML methods over GBLUP. Indeed, the predictive accuracies of SVR, KRR, RF, Adaboost.RT and GBLUP were 0.346, 0.349, 0.315, 0.349, and 0.290 respectively. In other words, ML methods improved the predictive accuracy by 12.8%, 14.9%, 5.4%, and 14.4%, respectively, over GBLUP. In summary, Liang et al. (2021) found a great advantage in using ML algorithms for GP in Simmental beef cattle, especially Adaboost.RT due to its reliability. However, the authors pointed out that ML models were sensitive to data, which means that two different datasets may have significant differences in predictive accuracy. Wang et al. (2022) used a pig dataset of 2566 Chinese Yorkshire pigs to compare the same models. The study concentrated on estimating the genomic breeding values of these individuals for two reproductive traits: the total number of piglets born (TNB) and the number of piglets born alive (NBA). The GEBVs were also estimated using classical methods [GBLUP, ssGBLUP, and Bayesian Horseshoe (BayesHE)]. Overall, ML methods outperformed conventional ones, and the degree of improvement over GBLUP, ssGBLUP, and BayesHE was 19.3%, 15.0% and 20.8% respectively. Furthermore, results showed that ML methods had the lowest MSE and MAE in all case scenarios. SVR and KRR provided the most consistent prediction abilities including higher accuracies and lower MSE and MAE. The findings of this study showed that ML methods are more efficient and had better performance in predicting GEBVs for reproductive traits, which can provide new insights for future GP. In another report, Sahebalam et al. (2019) evaluated the predictive ability of RF, SVM, the semiparametric model reproducing kernel Hilbert spaces (RKHS), and two parametric methods, namely, ridge regression and Bayes A. The ability of the above methods to predict was tested by estimating genomic breeding values for traits with different combinations of QTL effects, QTL numbers, three scenarios of heritability, and two training sets with 1,000 and 2,000 individuals. A genome of four chromosomes was simulated, and four generations were considered in the study. In the various simulation scenarios, the parametric methods outperformed semi-parametric (RKHS) and non-parametric ones (RF and SVM). However, the superiority of parametric models compared to semi-parametric ones was not statistically significant. In summary, Bayes A had the best prediction accuracy among all tested models.

Deep learning algorithms are found to be powerful in discovering intricate patterns and nonlinearity in large, messy datasets. Their application in genomic prediction has been investigated, however, the number of reports on DL application in animal breeding is small, and thus their potential should be further investigated. Gianola et al. (2011) evaluated the predictive ability of an artificial neural network to predict three quantitative traits, namely, milk, fat, and protein yield. In Jersey dairy cows. The dataset contained records of the milk yield of 297 Jersey dairy cows with a total of 35,798 SNPS. The authors conceived different Bayesian neural networks (BNN) with various architectures that differed in terms of the number of neurons, the type of activation function, and the source of the input variables, whether they were derived from pedigree or molecular markers. According to the results, BNNs with at least two neurons in the hidden layer had better performance. Moreover, results also showed that Bayesian regularization helped reduce the number of weights, which helped prevent overfitting. However, an overfitting problem still occurred in the Jersey training set, where large correlations between observed and predicted data were observed in the training set (0.90–0.95) and much lower correlations in the testing set. In another study, Beskorovajni et al. (2022) developed a multi-layer perceptron for predicting yield and fertility traits of 92 genotyped Holstein heifers, using several “Key traits” as input variables. These traits consist of Milk Yield, Fat Yield, Protein Yield, Somatic Cell Score (SCS), Productive Life (PL), Daughter Pregnancy Rate (DPR), Daughter Calving Ease (DCE), Final Type (PTA Type) and Genomic Future Inbreeding (GFI). An iterative method called the Broyden-Fletcher-Goldfarb-Shanno algorithm, which proceeds by minimizing the validation error, was used for optimization while training the ANN model. The authors obtained one optimal ANN for each target variable. The obtained ANN contained three layers, 11 neurons in the hidden layer and 276 weights and biases due to the high nonlinearity of the observed system. These hyper-parameters led to the highest values of r2 (0.951, 0.947, 0.989, 0.985, 0.902, 0.887, 0.676, 0.953, 0.590, 0.647, and 0.444) for these traits respectively; fat percentage, protein percentage, cheese merit, fluid merit, cow livability, sire calving ease, sire calving ease, heifer conception rate, cow conception rate, daughter stillbirth, sire stillbirth, and gestation length. In the end, Beskorovajni et al. (2022) found that the ANN (network MLP 9-11-11) based on the Broyden-Fletcher-Goldfarb-Shanno optimization algorithm did a good job of fitting the data and predicting yield and fertility traits. Waldmann et al. (2020) combined a one-dimensional CNN model with $l_{1}$ -norm regularization, Bayesian optimization and ensemble prediction within Genome Wide Prediction framework (CNNGWP) using simulated data with additive and dominance genetic effects and real pig data of 808 Australian Large White and Landrace sows with a total of 50174 SNPs. In comparison to findings achieved with GBLUP and the LASSO, the results demonstrate that CNNGWP does indeed reduce prediction error by more than 25% on simulated data and by about 3% on real pig data. In summary, Waldmann et al. (2020) pointed out that CNNGWP appears to offer a promising approach for GWP, however the degree of improvement depends on the genetic architecture and the heritability. A detailed guide about the implementation of DL for GP may be found in (Zingaretti et al., 2020).

In order to compare the performance of ensemble learning methods and deep learning algorithms, Abdollahi-Arpanahi et al. (2020), compared the performances of RF and GB with MLP and CNN, and two conventional tools, namely, GBLUP and Bayes B, in predicting quantitative traits using both simulated and real Holstein datasets. The simulated dataset was used to assess the performance of ML methods in different scenarios of genetic architectures. A quantitative trait was simulated and two scenarios of QTN number were considered: [small (100) and large (1,000)]. QTNs were located across the genome in two different ways: clustered or randomly, and gene action were either purely additive or a combination of additive, dominance and epistasis effects. On the other hand, real data from 11790 US Holstein bulls with a total of 57749 SNPs were used to test how well ML approaches can predict complex phenotypes like SCR, which is affected by both additive and non-additive effects. Abdollahi-Arpanahi et al. (2020) found that results differed depending on the genetic architecture of the trait. When pure additive actions controlled the trait, classic statistical models had better predictive accuracies compared to ML methods. However, the number of loci controlling the trait of interest appears to be an important factor in how well the models predicted outcomes when non-additive genetic effects occurred. The performance of ML algorithms, and in particular, GB, surpassed that of traditional statistical methods when the traits were controlled by a small number of QTN. The researchers finally came to the conclusion that, since Waldmann (2018) had already shown that loci are clustered, ML approaches work well for predicting traits with complex gene action and a small number of QTN (Abdollahi-Arpanahi et al., 2020).

Genomic prediction in animal breeding usually involves small reference population issues, especially when it concerns a novel trait, which can be costly and labor-intensive to measure. Machine learning models can be deployed to tackle these challenges. For example, Yao et al. (2016) developed a self-training model, which is a semi-supervised algorithm wrapped around SVM to encounter the challenge of genomic prediction of residual feed intake (RFI). The model uses 792 animals with both genotypes and phenotypes to train a base predictor, which is used to estimate the “self-trained phenotype” of 3,000 animals with genotypes only. To train a new predictor that is utilized to generate the final genomic predictions, both of these datasets are integrated. A total of 57491 SNPs were used for the analysis. The results showed that indeed, the self-training algorithm increased the accuracy of genomic prediction, however, this improvement was small when the dataset already contained more individuals with measured phenotypes. Additionally, the correlation between predicted and measured phenotypes increased by adding more self-trained phenotypes, however, it reached a plateau at a certain level. In summary, Yao et al. (2016) concluded that semi-supervised learning is a powerful tool for enhancing the accuracy of genomic prediction for novel traits and for small reference populations. However, choosing an adequate sample size and an adequate ML algorithm are necessary to prevent poor predictions. As an example, the predictive ability of RF models with a set-up similar to this study was assessed, and the authors found no improvement in accuracy from using self-training models (Yao et al., 2016).

### 5.2 Feature selection

Feature selection techniques are vital in genomic prediction. They allow us to identify the most informative genetic markers, mostly SNPs, that contribute to the traits of interest. In genomics, the massive amount of markers data poses a challenge in terms of computational efficiency and interpretability. By eliminating irrelevant markers, feature selection methods reduce noise and dimensionality, and increase the accuracy and performance of ML models. In addition, feature selection procedures enable the identification of key genetic variants, providing valuable insights into the biological mechanisms underlying traits of interest. Therefore, several studies have investigated the potential of ML models in performing feature selection using SNPs datasets of multiple animal species. Li et al. (2018a) applied three machine learning methods, namely, RF, GBM and XgBoost, for ranking the top 400, 1,000, and 3,000 SNPs directly related to the body weight of Brahman cattle to generate genomic relationship matrices (GRMs) for estimating genomic breeding values (GEBVs). The database used consisted of the body weight records of 2093 animals with a total of 38082 SNP markers. According to the results, RF and GBM outperformed XgBoost in identifying a subset of SNPs related to the growth trait. Furthermore, the top 3,000 SNPs identified by RF and GBM provided similar GEBV values to those of the whole SNP panel. In summary, the authors highly recommend the use of RF and GBM for identifying subsets of potential SNPs related to traits of interest. Besides, this approach could be very useful in animal breeding since the vast majority of research suffers from small reference population issues, whether it is due to genotyping cost constraints or to the nature of the target variable, which could be costly and labor-intensive to measure, such as feed efficiency. In this sense, Chen et al. (2021) compared the performance of two conventional methods, *t*-test and edgeR and three ensemble learning models, namely, RF, XGBoost, and a combination of both RF and XGBoost (RX) in identifying subsets of potential predictor genes in different tissues related to feed efficiency in Nellore Bulls. The dataset contained RNA sequences of five tissues (adrenal gland, hypothalamus, liver, skeletal muscle, and pituitary) from nine high-feed efficiency (HFE) and nine low-feed efficiency (LFE) bulls. Using the SVM model, the predictor genes that had been found using the above methods were used to divide the animals in the testing set into HFE and LFE. The performance of the classifier was evaluated using four metrics: overall accuracy, precision, recall and F1-score. The results showed that RX provided the best prediction accuracy yet with the smallest subset of genes (117). RF, in contrast, had the worst performance despite the fact that it had identified the largest number of candidate genes, contrary to what has been found in Naderi et al. (2016). The authors emphasize the idea that ML methods demonstrate great potential in identifying biologically relevant genes that can be used in classifying individuals accurately. In another study, Piles et al. (2021) implemented three types of feature selection methods: filter methods (tree-based methods), embedded methods (elastic net and LASSO regression), and a combination of both. Ridge regression, SVM, and GB were used after the pre-selection of relevant SNPs with filter methods. The results showed that using small subsets (50-250 SNPs), the feature selection method had a significant impact on prediction accuracy. In addition, filter methods demonstrated good performance and stability, indicating their potential for designing low density SNP chips for evaluating feed efficiency based on genomic information (Piles et al., 2021).

### 5.3 Genotype imputation

Genotype imputation plays a crucial role in animal genomics by inferring genotypes at specific positions in a genome by leveraging patterns and correlations within the data. Machine learning can be deployed to perform genotype imputation. For example, Sun et al. (2012) investigated the performance of Adaboost in imputing moderate-density genotypes from low-density panels in order to reduce genotyping costs. The proposed model works, in fact, by combining the imputation results of preexisting software packages. The database included 3059 registered genotyped Angus cattle and 51911 SNPs across the whole genome. The missing genotypes were first imputed by previously available packages, of which three were family-based and the others were population-based. Consequently, the possible combinations of the six packages resulted in 720 unique ensemble systems. The proposed Adaboost-based systems attribute a weight to each imputation method as a weak classifier. During the iterative training, the weights of classifiers that provided good predictions remained constant, whereas the weights of the misclassified samples were increased, which emphasized the focus on difficult samples. Finally, the final imputation of the genotype is the one with the majority of votes from all classifiers in the ensemble system. The results showed that indeed the ensemble method improved the accuracy of imputation in the data, however, the degree of improvement was limited by the fact that the packages used as weak classifiers had already provided highly accurate imputation results. Nevertheless, the authors highlighted the potential of ensemble learning to provide robust systems to address inconsistencies among different imputations of the preexisting methods.

## 6 Potential for ML applications to genomic prediction in animal breeding in developing countries

The majority of developing countries are grappling with satisfying the nutritional demands of an increasing human population. Meeting the demand for animal protein in a context of difficult environmental conditions and the predominance of smallholder systems in a sustainable manner is a challenging task. In addition, the introduction of highly productive dairy cows and the use of elite AI bulls’ semen to inseminate national dairy herds resulted in low productivity due to unfavorable genotypes by environment interaction. Moreover, it is delicate for developing countries to implement a consistent conventional genomic selection breeding scheme due to the lack of reliable phenotypes and pedigree data recording (Mrode et al., 2019). Therefore, in order to improve national livestock systems productivity, developing countries should find alternatives to the aforementioned bottlenecks. The development of genomic technologies and the remarkable decrease in genotyping costs can be valuable for low- and middle-income countries, as they can tackle pedigree error problems by using the genomic relationship matrix (G) instead of the relationship matrix (A) or combining both information in a matrix H. However, the size and structure of the reference population is the biggest struggle for adopting GS in developing countries, the number of genotyped animals is limited, usually between 500 and 3,000 animals, predominated by females due to the non-existence of AI bulls (Mrode et al., 2019). Collaborations with developed nations, as Li et al. (2016) describe, could therefore be advantageous for implementing GS in these nations. Also, the use of a mixture of high-density (HD) and low density (LD) chips followed by imputation to the HD could be an alternative for reducing even more the genotyping costs in order to increase the size of the reference population (Lashmar et al., 2019).

Considering indigenous breeds in breeding programs is indispensable in developing countries. First of all, the majority of smallholder systems’ dairy cows are either indigenous dairy cattle or crossbreds. Second, the conservation of genetic resources of local breeds that are adapted to specific agro-ecologies is crucial for the sustainability of the breed and biodiversity (Bulcha et al., 2022). Several countries, such as Kenya, Senegal, East Africa, Ethiopia, etc., have already implemented genomic technologies for indigenous breeds in Africa. Some studies used SNP data to determine the most adequate breed-type for different production environments. Others used genomic technologies to enhance breeding programs by increasing the accuracy of relationships among individuals. In other words, they have adopted genomic procedures to tackle the lack of pedigree recording. Finally, researchers investigated the potential of genomics for creating new breed-types that combine the adaptation and resilience of local breeds with the high productivity of exotic breeds. Genomic procedures and technologies have also been shown to be useful in discovering valuable genes in indigenous breed genomes, with significant effects due to the high levels of genome diversity of local breeds compared to exotic ones (Marshall et al., 2019).

Adopting GS in developing countries could benefit from the implementation of machine learning algorithms. First of all, given that indigenous breeds always have small reference populations, machine learning has shown great advantage in increasing the accuracy of breeding values estimation in small populations, as previously seen in Yao et al. (2016). In addition, ML models increased the accuracy of SNP imputation from low-density (LD) panels to high density (HD) chips, as investigated by Sun et al. (2012). This could result in reducing genotyping costs and increasing the size of genotyped animals (if the reference population is small due to genotyping costs). Overall, the potential of applying machine learning models for animal breeding in low- and medium-income countries is remarkable, as it could provide insightful findings. However, one of the biggest challenges would be the lack of data. Machine learning models typically require a massive amount of data in order to achieve high accuracy, while low- and middle-income countries often struggle with limited access to reliable data. Nonetheless, efforts should be directed toward exploring alternative techniques to enhance genomic prediction accuracy using a small reference population and promoting data sharing through collaborations among institutes and countries. As far as we know, the combination of machine learning models and genomic prediction in developing countries has not been used in any of the published studies, and thus their potential in enhancing breeding programs in low- and middle-income countries should be investigated in future experiments.

## 7 Conclusion

Machine learning algorithms have proven their high flexibility and ability to extract patterns in large, messy datasets in various fields such as natural language processing, robotics, speech recognition, image processing, etc. Genomic prediction is indeed a field of study where the main challenge is dealing with an ever-increasing marker dataset and capturing interactions and non-additive effects between genotypes. Consequently, investigating the potentiality of ML algorithms in GP is gaining a lot of buzz in the animal breeding community. Here, we reviewed studies that applied ML models to GP, whether they concerned estimating the GEBVs for production traits, health traits, or novel traits. In addition, several studies used ML algorithms for feature selection (FS) and moderate-density genotype imputation from low-density panels. It can be observed that ML algorithms outperformed conventional methods in some studies but were less accurate in others, which indicates that there’s no universal method that can be applied to enhance the accuracy of prediction regardless of the domain of application. As a prerequisite, one should pay attention to several factors in order to successfully apply ML algorithms. For instance, the nature of the task, whether it consists of classification, clustering, regression, or dimensionality reduction, the type of the target variable (continuous or discrete), and the quality of the data (redundant, noisy, existence of outliers, missing values). ML models are indeed flexible and powerful, but they also have several drawbacks. One of the most common problems encountered in ML is overfitting. Additionally, finding the optimal hyperparameters can be challenging, and the size of the training data needs to be very large, especially for training deep learning algorithms. It is indeed true that incorporating ML algorithms and biological knowledge provides valuable results. However, marker datasets tend to be very heterogeneous and redundant, which can lower the predictive ability of these models. Moreover, the interpretability of non-parametric ML models is also questionable. Even though the algorithm’s prediction for a particular target variable is accurate, the relationship between the input and output variables is not simple to understand. In fact, DL models are broadly known for their “Black Box” nature, which means that their interpretation cannot extract relevant information about variables in the dataset. In summary, ML algorithms showed great potential for fitting and extracting patterns from large, noisy datasets. However, their adoption in livestock breeding is still in its infancy, and hence more research must be done in order to find new insights for GP. The limited number of applications of ML in animal breeding did not allow researchers to clarify the huge potential for these models to improve the genomic prediction of important traits. Therefore, more iterative experimentation needs to be conducted.
