# Supplementary material for: A review of machine learning models applied to genomic prediction in animal breeding
Source: Front Genet. 2023 Sep 6;14:1150596. doi: 10.3389/fgene.2023.1150596 (PMC10516561; doi:10.3389/fgene.2023.1150596)
Supplement: Supplementary file 1 [file DataSheet1.pdf]

Supplementary Material

A review of machine learning models applied to genomic prediction in animal breeding

Narjice Chafai[1], Ichrak Hayah[1], Isodore Houaga[2,3], Bouabid Badaoui[1,4]*

Correspondence:

bouabidbadaoui@gmail.com

1 Supplementary Tables

Table 1 Machine learning models applied to genomic prediction in animal breeding

Year	Authors	Species	Breed	No. of individuals	No. of markers	Response variable	ML algorithms	Aim of the study
**2011**	Gianola et al.	Dairy cattle	Jersey	297	35798 SNPs	MY, MFY, MPY	BNN	Investigating the predictive ability of ANN using a Bayesian regularization in analyzing complex traits.
**2012**	Sun et al.	Beef cattle	Angus	3059	51911 SNPs	Imputing 50K genotypes from 5K genotypes	Adaboost	Proposing an Adaboost-like approach for the imputation of moderate-density genotypes from low-density panels.
**2015**	Ehret et al.	Dairy cattle	Holstein and Fleckvih	218	41423 SNPs	the concentration of BHBA in milk.	ANN (MLP)	Applying an ANN approach to investigate the utility of metabolic, genetic, and milk performance data for predicting milk levels of BHBA within and across weeks of postpartum.
**2016**	Naderi et al.	Dairy cattle (simulated)		20000 females and 400 males	50025 and 10005 SNPs	Subclinical Ketosis	ANN (MLP)	Building an ANN for an earlier prediction of subclinical Ketosis in lactation.
**2016**	Mikshowsky et al.	Dairy cattle	Holstein	17276	57169 SNPs	MPY, SCS, DPR	Bagging	Evaluate the performance of bagging GBLUP in identifying bulls whose daughter performance is more likely to deviate from early genomic prediction.
**2016**	Yao et al.	Dairy cattle	Holstein	3000 genotyped 792 genotyped and phenotyped	57491 SNPs	RFI	SVM (semi-supervised learning)	Describing a SVM-based semi-supervised learning model, and applying it for genomic prediction of residual feed intake.

**2018**	Waldmann	Simulated data		-	-	Five anonymous traits	ABNN	Proposing an approximate Bayesian Neural network ABNN model for large genomic data that can be modified easily.
		Pig	Large White and Landrace	3534	52842 SNPs	A continuous quantitative trait		
**2018**	Li et al.	Beef cattle	Brahman	2093	40184	BW	RF, GBM, XGBoost	Assessing the efficiency of three ML methods in identifying the top-ranked SNPs and using the subsets of SNPs to construct genomic relationship matrices for estimating genomic breeding values.
**2020**	Liang et al.	Beef cattle	Simmental	1217	671900 SNPs	CW, LW, EMA	Adaboost.RT (integrated SVR), KRR, RF	Applying ensemble learning models to predict genomic breeding values of three economic traits.
**2020**	Abdollahi-Arpanahi et al.	Dairy cattle	Holstein	1170	57749 SNPs	SCR	MLP, CNN, RF, GB	Comparing the predictive performance of two deep learning methods, two ensemble learning methods and two parametric methods (GBLUP and Bayes B).
		Simulated data		-	100 and 1000 QTNs	A quantitative trait		
**2021**	Chen et al.	Beef cattle	Nellore	18	16,423 genes	FE	RF, XGBoost, RX, SVM	Applying Rf, XGBoost and RX to identify small subsets of biologically important genes to classify animals into High Feed Efficiency and Low Feed Efficiency.
**2021**	Srivastava et al.	Beef cattle	Hanwoo	7324	53866 SNPs	CWT, MS, BFT EMA	RF, XGB, SVM	Comparing the predictive ability of three ML models in predicting phenotypes from genotypes.
**2021**	An et al.	Beef cattle	Simmental	1301	671990 SNPs	LW, CW, EMA	KcRR, SVR	Assessing the prediction accuracies of 12 traits with various heritabilities and genetic architectures using parametric methods (GBLUP and Bayes B), and two machine learning models (KcRR and SVR)
		Dairy cattle	Holstein	5024	42551 SNPs	MY, MFP, SCS		
		Pig		3534	43494, 43407, and 43412 SNPs	T1, T2, T3		

Supplementary Material

**2021**	Wang et al.	Pig	Yorkshire	2566	44922 SNPs	TNB, NBA	SVR, KRR, RF, Adaboost.R2	Exploring and comparing the prediction ability of four ML models to GBLUP, ssGBLUP and bayesian methods in genomic prediction of reproductive traits.
**2021**	Beskorovajni et al.	Dairy cattle	Holstein	92	-	MFP, MPP, CM, FM, LIV, SCE, HCR, CCR, DSB, SSB, GL	MLP	Predicting yield and fertility traits using an MLP model based on the Broyden-Fletcher-Goldfarb-Shanno iterative optimization algorithm for genomic selection.

Table 2 Programming languages and packages used to run machine learning algorithms applied in the reviewed studies

Published paper reference	ML algorithms used	Programming language	Package/library	Library's reference
Ogutu et al. (2011)	RF	R	Random forest	Liaw and Wiener (2002)
	SGB		Gbm	Ridgeway G: Gbm: Generalized boosted regression models. R package, version 1.6-3.1. Available at http://cran.r-project.org/web/packages/gbm/.
	SVMs		e1071	Dimitriadou et al. (2005)
Gianola et al. (2011)	BNN	MATLAB	Neural networks toolbox	Beal, Hagan, and Demuth HB (2010)
González-Recio and Forni (2011)	RF	Java	-	-
Ehret et al. (2015)	ANN (MLP)	C++	-	-
Naderi et al. (2016)	RF	Java	RanFoG	González-Recio and Forni, (2011)
Mikshowsky et al.(2017)	Bagging GBLUP	-	-	-
	BGLR	R	BGLR	-
Yao et al. (2016)	SVM	R	e1071	Dimitriadou et al. (2005)
Waldmann (2018)	BNN	Python	MXNet	Chen et al. (2015)
Li et al. (2018)	RF	R	randomForest	Liaw and Wiener (2002)
	GBM		GBM	Ridgeway G: Gbm: Generalized boosted regression models. R package, version 1.6-3.1. Available at http://cran.r-project.org/web/packages/gbm/.
	XGBoost		Xgboost	Chen et al. (2017)
Liang et al. (2020)	Adaboost.RT	Python	Sklearn V0.22	-
	KRR			

	RF			
	SVM			
Zhao et al. (2020)	SVM	Python	Scikit-learn	Pedregosa et al. (2012)
Liang et al. (2020)	Adaboost.RT	Python	Sklearn V0.22	-
	KRR			
	RF			
	SVM			
Liang et al. (2020)	Adaboost.RT	Python	Sklearn V0.22	-
Waldmann et al. (2020)	CNNGWP	R	R interface to Keras with theTensorFlow back-end	-
Abdollahi-Arpanahi et al.(2020)	MLP	R	MXNet	Chen et al. (2015)
	CNN		DeepGS	Ma et al. (2018)
	RF		randomForest	Liaw and Wiener (2002)
	XGBoost		-	Chen at al. (2015)
Chen et al. (2021)	RF	R	randomForest	Liaw and Wiener (2002)
	XGBoost		XGBoost	Chen et al. (2015)
	RX		randomForest and XGBoost	Liaw and Wiener (2002) ; Chen et al. (2015)
	SVM		e1071	Meyer et al. (2019)
An et al. (2021)	KcRR	Python	Sklearn V0.22	-
Wang et al. (2021)	SVR	Python	Sklearn V0.22	-
	KRR			
	RF			
	Adaboost.R2			
Srivastava et al. (2021)	RF	Python	Scikit-learn	Pedregosa et al. (2012)
	XGBoost			
	SVM			

References

Abdollahi-Arpanahi, R., Gianola, D., & Peñagaricano, F. (2020). Deep learning versus parametric and ensemble methods for genomic prediction of complex phenotypes. *Genetics Selection Evolution*, *52*, 1-15. https://doi.org/10.1186/s12711-020-00531-z.

An, B., Liang, M., Chang, T., Duan, X., Du, L., Xu, L., Zhang, L., Gao, X., Li, J., & Gao, H. (2021). KCRR: A Nonlinear Machine Learning with a Modified Genomic Similarity Matrix Improved the Genomic Prediction Efficiency. *Briefings in Bioinformatics* 22 (6): bbab132. https://doi.org/10.1093/bib/bbab132.

Beale, M. H., Hagan, M. T., & Demuth, H. B. (2010). Neural network toolbox. *User's Guide, MathWorks*, *2*, 77-81.

Beskorovajni, R., Jovanović, R., Pezo, L., Popović, N., Tolimir, N., Mihajlović, L., & Šurlan-Momirović, G. (2022). Mathematical modeling for genomic selection in Serbian dairy cattle. *Genetika*, *53*(3), 1105-1115. https://doi.org/10.2298/GENSR2103105B.

Chen, T., He, T., and Benesty, M. (2016). Xgboost: Extreme Gradient Boosting. Available online at: https://cran.r-project.org/web/packages/xgboost/vignettes/xgboost.pdf (accessed January 5, 2021).

Chen T, Li M, Li Y, Lin M, Wang N, Wang M. (2017). MXNet: a flexible and efficient library for deep learning. https ://mxnet .incubator.apach e.org/.

Chen, T., Li, M., Li, Y., Lin, M., Wang, N., Wang, M., Xiao, T., Xu, B., Zhang, C., Zhang, Z. (2015). MXNet: A Flexible and Efficient Machine Learning Library for Heterogeneous Distributed Systems (No. arXiv:1512.01274). arXiv. https://doi.org/10.48550/arXiv.1512.01274

Chen, W., Alexandre, P. A., Ribeiro, G., Fukumasu, H., Sun, W., Reverter, A., & Li, Y. (2021). Identification of predictor genes for feed efficiency in beef cattle by applying machine learning methods to multi-tissue transcriptome data. *Frontiers in Genetics*, *12*, 619857. https://doi.org/10.3389/fgene.2021.619857.

Dimitriadou, E., Hornik, K., Leisch, F., Meyer, D., & Weingessel, A. (2008). Misc functions of the Department of Statistics (e1071), TU Wien. *R package*, *1*, 5-24. http://CRAN.R-project.org/.

Supplementary Material

Ehret, A., D. Hochstuhl, N. Krattenmacher, J. Tetens, M.S. Klein, W. Gronwald, and G. Thaller. (2015). Short Communication: Use of Genomic and Metabolic Information as Well as Milk Performance Records for Prediction of Subclinical Ketosis Risk via Artificial Neural Networks. *Journal of Dairy Science* 98 (1): 322–29. https://doi.org/10.3168/jds.2014-8602.

Gianola, D., Okut, H., Weigel, K. A., & Rosa, G. J. (2011). Predicting complex quantitative traits with Bayesian neural networks: a case study with Jersey cows and wheat. *BMC genetics*, *12*, 1-14.

González-Recio, O., & Forni, S. (2011). Genome-wide prediction of discrete traits using Bayesian regressions and machine learning. *Genetics Selection Evolution*, *43*, 1-12.

Li, B., Zhang, N., Wang, Y. G., George, A. W., Reverter, A., & Li, Y. (2018). Genomic prediction of breeding values using a subset of SNPs identified by three machine learning methods. *Frontiers in genetics*, *9*, 237. https://doi.org/10.3389/fgene.2018.00237.

Liang, M., Miao, J., Wang, X., Chang, T., An, B., Duan, X., Xu, L., Gao, X., Zhang, L., Li, J., & Gao, H. (2021). Application of ensemble learning to genomic selection in chinese simmental beef cattle. *Journal of Animal Breeding and Genetics*, *138*(3), 291-299. https://doi.org/10.1111/jbg.12514.

Liaw, A., & Wiener, M. (2002). Classification and regression by randomForest. *R news*, *2*(3), 18-22. https://cogns.northwestern.edu/cbmg/LiawAndWiener2002.pdf.

Ma, W., Qiu, Z., Song, J., Li, J., Cheng, Q., Zhai, J., & Ma, C. (2018). A deep convolutional neural network approach for predicting phenotypes from genotypes. *Planta*, *248*, 1307-1318.https://doi.org/10.1007/s00425-018-2976-9

Meyer, D., Dimitriadou, E., Hornik, K., Weingessel, A., Leisch, F., Chang, C. C., & Lin, C. (2015). Misc functions of the Department of Statistics. *Probability Theory Group (Formerly: E1071), TU Wien*. https://cran.microsoft.com/snapshot/2016-08-05/web/packages/e1071/index.html.

Meyer, D., Dimitriadou, E., Hornik, K., Weingessel, A., Leisch, F., Chang, C. C., & Lin, C. C. (2019). e1071: misc functions of the department of statistics, probability theory group (formerly: E1071), TU Wien. *R package version*, *1*(2).

Mikshowsky, A. A., Gianola, D., & Weigel, K. A. (2017). Assessing genomic prediction accuracy for Holstein sires using bootstrap aggregation sampling and leave-one-out cross validation. *Journal of Dairy Science*, *100*(1), 453-464. https://doi.org/10.3168/jds.2016-11496.

Naderi, S., Yin, T., & König, S. (2016). Random forest estimation of genomic breeding values for disease susceptibility over different disease incidences and genomic architectures in simulated cow calibration groups. *Journal of Dairy Science*, *99*(9), 7261-7273. https://doi.org/10.3168/jds.2016-10887.

Ogutu, J. O., Piepho, H. P., & Schulz-Streeck, T. (2011, December). A comparison of random forests, boosting and support vector machines for genomic selection. In *BMC proceedings* (Vol. 5, No. 3, pp. 1-5). BioMed Central. https://doi.org/10.1186/1753-6561-5-S3-S11.

Pedregosa, F., Varoquaux, G., Gramfort, A., Michel, V., Thirion, B., Grisel, O., Blondel, M., Prettenhofer, P., Weiss, R., Dubourg, V., Vanderplas, J., Passos, A., Cournapeau, D., Brucher, M., Perrot, M., Duchesnay, E., Louppe, G. (2012). Scikit-learn: Machine Learning in Python. J. Mach. Learn. Res. 12.

Ridgeway G: Gbm: Generalized boosted regression models. R package, version 1.6-3.1. Available at http://cran.r-project.org/web/packages/gbm/.

Srivastava, S., Lopez, B. I., Kumar, H., Jang, M., Chai, H. H., Park, W, Park, J. E.,& Lim, D. (2021). Prediction of Hanwoo cattle phenotypes from genotypes using machine learning methods. *Animals*, *11*(7), 2066. 2066. https://doi.org/10.3390/ani11072066.

Sun, C., Wu, X. L., Weigel, K. A., Rosa, G. J., Bauck, S., Woodward, B., Schnabel R. D., Taylor, J. F., & Gianola, D. (2012). An ensemble-based approach to imputation of moderate-density genotypes for genomic selection with application to Angus cattle. *Genetics research*, *94*(3), 133-150.. https://doi.org/10.1017/S001667231200033X.

Wang, X., Shi, S., Wang, G., Luo, W., Wei, X., Qiu, A., Luo, F., & Ding, X. (2022). Using machine learning to improve the accuracy of genomic prediction of reproduction traits in pigs. *Journal of Animal Science and Biotechnology*, *13*(1), 1-12. https://doi.org/10.21203/rs.3.rs-1083849/v1.

Waldmann, P. (2018). Approximate Bayesian neural networks in genomic prediction. *Genetics Selection Evolution*, *50*, 1-9. https://doi.org/10.1186/s12711-018-0439-1.

Waldmann, P., Pfeiffer, C., & Mészáros, G. (2020). Sparse convolutional neural networks for genome-wide prediction. *Frontiers in Genetics*, *11*, 25. https://doi.org/10.3389/fgene.2020.00025.

Supplementary Material

Yao, C., Zhu, X., & Weigel, K. A. (2016). Semi-supervised learning for genomic prediction of novel traits with small reference populations: an application to residual feed intake in dairy cattle. *Genetics Selection Evolution*, *48*, 1-9. https://doi.org/10.1186/s12711-016-0262-5.

Zhao, W., Lai, X., Liu, D., Zhang, Z., Ma, P., Wang, Q., Zhang, Z., & Pan, Y. (2020). Applications of support vector machines in genomic prediction in pig and maize populations. *Frontiers in genetics*, *11*, 598318.https://doi.org/10.3389/fgene.2020.598318.
